# Comorbidity of dementia: a cross-sectional study of primary care older patients

**DOI:** 10.1186/1471-244X-14-84

**Published:** 2014-03-20

**Authors:** Beatriz Poblador-Plou, Amaia Calderón-Larrañaga, Javier Marta-Moreno, Jorge Hancco-Saavedra, Antoni Sicras-Mainar, Michael Soljak, Alexandra Prados-Torres

**Affiliations:** 1 EpiChron Research Group on Chronic Diseases, Aragón Health Sciences Institute (IACS), IIS Aragón, Miguel Servet University Hospital, Zaragoza, Spain; 2 Red de Investigación en Servicios de Salud en Enfermedades Crónicas (REDISSEC), Carlos III Health Institute, Madrid, Spain; 3 Teaching Unit of Preventive Medicine and Public Health, Aragón Health Sciences Institute (IACS), IIS Aragón, Zaragoza, Spain; 4 Department of Microbiology, Preventive Medicine and Public Health, University of Zaragoza, Zaragoza, Spain; 5 Miguel Servet University Hospital Department of Neurology, Zaragoza, Spain; 6 Planning Management, Badalona Serveis Assistencials S.A, Badalona, Spain; 7 Department of Primary Care & Public Health, Imperial College London, London, UK

**Keywords:** Dementia, Comorbidity, Factor analysis, Primary care, Electronic health records

## Abstract

**Background:**

The epidemiologic study of comorbidities of an index health problem represents a methodological challenge. This study cross-sectionally describes and analyzes the comorbidities associated with dementia in older patients and reviews the existing similarities and differences between identified comorbid diseases using the statistical methods most frequently applied in current research.

**Methods:**

Cross-sectional study of 72,815 patients over 64 seen in 19 Spanish primary care centers during 2008. Chronic diseases were extracted from electronic health records and grouped into Expanded Diagnostic Clusters®. Three different statistical methods were applied (i.e., analysis of prevalence data, multiple regression and factor analysis), stratifying by sex.

**Results:**

The two most frequent comorbidities both for men and women with dementia were hypertension and diabetes. Yet, logistic regression and factor analysis demonstrated that the comorbidities significantly associated with dementia were Parkinson’s disease, congestive heart failure, cerebrovascular disease, anemia, cardiac arrhythmia, chronic skin ulcers, osteoporosis, thyroid disease, retinal disorders, prostatic hypertrophy, insomnia and anxiety and neurosis.

**Conclusions:**

The analysis of the comorbidities associated with an index disease (e.g., dementia) must not be exclusively based on prevalence rates, but rather on methodologies that allow the discovery of non-random associations between diseases. A deep and reliable knowledge about how different diseases are grouped and associated around an index disease such as dementia may orient future longitudinal studies aimed at unraveling causal associations.

## Background

Dementia is characterized by the impairment of memory and learning and at least one other cognitive domain (i.e., aphasia, apraxia, agnosia or executive function), representing a highly severe functional deterioration that interferes with the patient’s daily functional abilities and independence [1]. Dementia is not simply a disease; it is a syndrome caused by different etiologies and it has a substantial medical and social impact. Although recent door-to-door studies suggest a decreasing incidence of dementia [2,3], its crude prevalence has increased continuously over the past decades due to aging populations. The World Health Organisation (WHO) estimated that the number of persons living with dementia worldwide (36 million in 2010) will double over the next 20 years [4]. According to the World Alzheimer Report from 2013 [5], individuals afflicted with dementia show a high utilization of health services and represent a significant fraction of the healthcare costs attributed to the elderly population. In fact, dementia currently constitutes the main cause of dependence in the elderly population and is responsible for up to 12% of the years lived with disability due to a non-communicable disease [6].

Moreover, it is known that patients with dementia have on average 2 to 8 additional chronic diseases (comorbidities) [7,8]. They may accelerate progression towards a state of cognitive and functional impairment that results in the under-diagnosis and under-treatment of dementia [9]. In addition, these comorbidities lead to extended hospital stays and increased healthcare costs and mortality rates for hospitalized patients [10]. As is true for other clinically complex circumstances, the presence of comorbidities in patients with dementia requires us to consider patients from a global perspective [11], prioritizing certain health and health outcomes over others, and taking into account possible conflicts between multiple treatments and recommendations for these patients [12].

The epidemiologic study of comorbidities of an index diagnosis (dementia, in this case) is complex since, as Ording et al. recently indicated [13], concepts such as comorbidity and complications are often difficult to differentiate. Furthermore, a cross-sectional vs. life-course study design can have an important impact on the direction of the identified associations, as highlighted in the Neurological Disorders in Central Spain (NEDICES) Study [14,15]. Still, cross-sectional studies provide a useful perspective to build future longitudinal and more specific approaches, especially if techniques that enable the identification of beyond-chance associations are employed [16].

Improved knowledge of the comorbidities of highly prevalent chronic health problems, such as dementia, would facilitate the design of preventive strategies aimed at slowing or avoiding the rapid clinical and functional deterioration which afflicts patients with this index disease [17]. Moreover, it may help overcome the potential undertreatment of those disorders that are not designated as the primary condition [18].

This study cross-sectionally describes and analyzes the comorbidities associated with dementia and reviews the existing similarities and differences between identified comorbid diseases using the statistical methods most frequently applied in current research.

## Methods

### Study design and participants

A cross-sectional study was performed based on electronic health record data from primary care. The study population comprised individuals 65 years of age and older who, in the year 2008, consulted their primary care physician at least once at any of the 19 primary healthcare centers included in this study. The centers were located in two regions of Spain: Aragon and Catalonia. The selection and data-quality criteria for the participating centers were described elsewhere [19].

The analyzed patient data were age, sex and chronic diagnoses. To facilitate the use of clinical information, diagnoses were grouped according to the Expanded Diagnostic Clusters (EDC) of the ACG® system. To this purpose, each diagnosis contained in the electronic health records and originally coded according to the International Classification of Primary Care (ICPC) was previously transformed into the International Classification of Diseases (ICD-9-CM). Finally, the ACG® system grouped each ICD-9-CM code into one of 264 EDCs based on the clinical, diagnostic and therapeutic similarities of the diseases.

The selection of chronic EDCs was based on a previous study conducted by Salisbury et al. [20], in which a chronic disease was defined as “one that normally lasts 6 months or more, including past conditions that require ongoing disease or risk management. They must be important conditions with a significant risk of recurrence, or past conditions that have continuing implications for patient management”. Dementia was defined using the EDC NUR11 category “dementia and delirium”, once delirium diagnoses were excluded.

This study was favorably evaluated by the Clinical Research Ethic Committee of Aragon (CEICA). Written consent by patients was not needed since the work is based on the statistical analysis of anonymous data contained in previously existing databases.

### Statistical analysis

Three statistical methods were applied. The first was based on the analysis of prevalence data, and the remaining two were based on multivariate analysis techniques (i.e., multiple logistic regression and exploratory factor analysis).

1) Analysis of prevalence

The prevalence of chronic comorbidities associated with dementia was calculated for both men and women. To identify the most prevalent associations, only combinations of two diseases were considered (e.g., dementia and a comorbidity), discarding higher rank-order combinations (e.g., triads and tetrads).

2) Multiple logistic regression models

A multiple logistic regression model was formulated stratifying by sex and assuming the presence or absence of dementia as a dependent variable. The remaining chronic diseases with prevalences equal to or greater than 1% were considered as covariates for each group under study. The selection of variables for inclusion in the model was based on the backward elimination procedure with an initial inclusion probability of p < 0.05 and an exclusion probability of p ≥ 0.1.

3) Exploratory factor analysis

Exploratory factor analysis is based on the design of a mathematical model with the capability of explaining correlations (covariance) between high numbers of observed variables and reducing them to a lower number of latent variables, known as factors [21]. The objective is the identification of sets of variables with an underlying common causal factor. This method, in addition to identifying associations between groups of variables, allows the same variable to become part of several factors.

Similar to the logistic regression models, only diseases with a prevalence of ≥1% were included for each study group. Factor analysis was based on tetrachoric correlation matrices [22], and the factors were extracted using the principal factor method. The number of factors to be extracted was determined using scree plots [23] and the clinical evaluation of the different solutions obtained. The sampling adequacy was analyzed using the Kaiser-Meyer-Olkin (KMO) parameter, and the cumulative fraction of total variance was used as a measure of the model’s goodness of fit. EDCs with a factor score higher than 0.25 were selected, with the aim of determining the diseases that composed each pattern.

STATA 11.0 software was used for the statistical analyses, and Excel 2007 was used to produce the corresponding graphs.

## Results

The studied population consisted of 72,815 patients, of whom 3,971 (5.45%) were diagnosed with dementia. The frequency of dementia in women was more than double that in men (Table 1). Patients with dementia were, on average, four years older than those without the index disease (80 vs. 76 years). Among the patients with dementia, 12.34% had dementia as the only diagnosis, 69.61% showed at least two comorbidities and 48.05% showed at least three. The average number of comorbidities in the population with dementia (3.69 in men and 3.68 in women) was significantly higher than in the population not diagnosed with dementia (2.32 in men and 2.52 in women).

**Table 1:** Study population

	**Patients ≥65 without dementia**			**Patients ≥65 with dementia**			**p value**
	**Total**	**Men**	**Women**	**Total**	**Men**	**Women**	
**n (%)**	68,844 (94.55)	28,176 (40.93)	40,668 (59.07)	3,971 (5.45)	1,185 (29.84)	2,786 (70.16)	0.000
**Mean age (SD)**	75.53 (7.28)	74.62 (6.85)	76.16 (7.51)	80.22 (7.09)	79.10 (6.94)	80.70 (7.10)	0.000
**Number of diseases n (%)**							
**1**	15,052 (21.86)	6,718 (23.84)	8,334 (20.49)	490 (12.34)*	137 (11.56)*	353 (12.67)*	0.000
**2**	15,934 (23.15)	6,730 (23.89)	9,204 (22.63)	717 (18.06)	218 (18.40)	499 (17.91)	0.000
**3**	12,795 (18.59)	5,085 (18.05)	7,710 (18.96)	856 (21.56)	260 (21.94)	596 (21.39)	0.000
**4**	8,500 (12.35)	3,203 (11.37)	5,297 (13.02)	700 (17.63)	222 (18.73)	478 (17.16)	0.000
**5**	4,604 (6.69)	1,634 (5.80)	2,970 (7.30)	555 (13.98)	151 (12.74)	404 (14.50)	0.000
**≥6**	3,831 (5.55)	1,320 (4.68)	2,511 (6.17)	653 (16.44)	197 (16.62)	456 (16.38)	0.000
**Mean number of diseases (SD)**	2.44 (1.75)	2.32 (1.69)	2.52 (1.79)	3.69 (1.95)	3.69 (1.94)	3.68 (1.96)	0.000

SD, standard deviation.

*Patients with dementia only.

### Prevalence of comorbidities

A total of 43 different comorbidities with a prevalence of ≥1% were identified in the population with dementia (41 different comorbidities in men and 36 in women). The 10 diseases with the highest prevalence for both sexes were hypertension, anxiety and neurosis, degenerative joint disease, lipid metabolism disorders, lower back pain, diabetes, anemia, thyroid disease, cataracts and aphakia, and cardiac arrhythmia (Table 2).

**Table 2:** Prevalence (95% CI) of chronic comorbidities of dementia in ≥65-year-old men and women

**Rank**	**Disease**	**Men**	**Disease**	**Women**
**1**	Hypertension	38.6 (35.9 - 41.4)	Hypertension	44.9 (43.1 - 46.8)
**2**	Diabetes	20.3 (18.0 - 22.5)	Anxiety, neuroses	23.4 (21.9 - 25.0)
**3**	Prostatic hypertrophy	19.0 (16.8 - 21.2)	Degenerative joint disease	20.5 (19.0 - 22.0)
**4**	Degenerative joint disease	15.3 (13.2 - 17.3)	Disorders of lipid metabolism	18.7 (17.2 - 20.1)
**5**	Disorders of lipid metabolism	15.3 (13.2 - 17.3)	Low back pain	16.6 (15.2 - 18.0)
**6**	Low back pain	14.1 (12.1 - 16.1)	Diabetes	15.8 (14.4 - 17.1)
**7**	Anxiety, neuroses	13.8 (11.9 - 15.8)	Osteoporosis	10.9 (9.8 - 12.1)
**8**	COPD	11.0 (9.2 - 12.8)	Anemia	9.8 (8.7 - 10.9)
**9**	Cardiac arrhythmia	9.5 (7.9 - 11.2)	Thyroid disease	8.4 (7.3 - 9.4)
**10**	Dermatitis and eczema	9.4 (7.7 - 11.0)	Varicose veins of lower extremities	7.4 (6.4 - 8.3)
**11**	Anemia	9.1 (7.5 - 10.8)	Cataracts, aphakia	7.1 (6.1 - 8.0)
**12**	Cataracts, aphakia	6.8 (5.3 - 8.2)	Cardiac arrhythmia	6.9 (5.9 - 7.8)
**13**	Cerebrovascular disease	6.7 (5.2 - 8.1)	Dermatitis and eczema	6.9 (5.9 - 7.8)
**14**	Ischemic heart disease^1^	6.6 (5.2 - 8.0)	Chronic skin ulcers	4.7 (3.9 - 5.5)
**15**	Thyroid disease	4.5 (3.3 - 5.7)	Cerebrovascular disease	4.6 (3.8 - 5.3)
**16**	Generalized atherosclerosis	4.2 (3.1 - 5.4)	Gastroesophageal reflux	3.9 (3.2 - 4.6)
**17**	Malignant neoplasms, prostate	4.1 (3.0 - 5.3)	Congestive heart failure	3.8 (3.1 - 4.5)
**18**	Glaucoma	4.0 (2.9 - 5.1)	Glaucoma	3.7 (3.0 - 4.4)
**19**	Gastroesophageal reflux	3.4 (2.3 - 4.4)	Ischemic heart disease^1^	3.3 (2.6 - 4.0)
**20**	Cervical pain syndromes	3.4 (2.3 - 4.4)	Behavior problems	3.1 (2.5 - 3.8)
**21**	Parkinson's disease	3.0 (2.0 - 3.9)	Cervical pain syndromes	3.1 (2.4 - 3.7)
**22**	Chronic skin ulcers	2.9 (1.9 - 3.8)	Asthma	2.6 (2.0 - 3.2)
**23**	Congestive heart failure	2.8 (1.8 - 3.7)	Low-impact malignant neoplasms	2.6 (2.0 - 3.2)
**24**	Acute myocardial infarction	2.8 (1.8 - 3.7)	Obesity	2.6 (2.0 - 3.2)
**25**	Varicose veins of lower extremities	2.5 (1.6 - 3.4)	Cardiovascular disorders, other	2.5 (1.9 - 3.1)
**26**	Hematologic disorders, other	2.4 (1.5 - 3.2)	COPD	2.5 (1.9 - 3.1)
**27**	Deafness, hearing loss	2.4 (1.5 - 3.2)	Hematologic disorders, other	2.2 (1.7 - 2.8)
**28**	Osteoporosis	2.0 (1.2 - 2.8)	Deafness, hearing loss	2.0 (1.5 - 2.6)
**29**	Low-impact malignant neoplasms	2.0 (1.2 - 2.8)	Peripheral neuropathy, neuritis	2.0 (1.5 - 2.5)
**30**	Obesity	1.9 (1.1 - 2.6)	Generalized atherosclerosis	1.8 (1.3 - 2.3)
**31**	Cardiovascular disorders, other	1.8 (1.0 - 2.5)	Depression	1.6 (1.1 - 2.0)
**32**	Behavior problems	1.7 (1.0 - 2.4)	Parkinson's disease	1.5 (1.1 - 2.0)
**33**	Gout	1.6 (0.9 - 2.3)	Diverticular disease of colon	1.4 (1.0 - 1.8)
**34**	Malignant neoplasms, colorectal	1.5 (0.8 - 2.2)	Other endocrine disorders	1.3 (0.9 - 1.7)
**35**	Retinal disorders^2^	1.4 (0.7 - 2.0)	Thrombophlebitis	1.3 (0.9 - 1.7)
**36**	Peripheral neuropathy, neuritis	1.2 (0.6 - 1.8)	Acute myocardial infarction	1.1 (0.8 - 1.5)
**37**	Renal calculi	1.2 (0.6 - 1.8)		
**38**	Thrombophlebitis	1.1 (0.5 - 1.7)		
**39**	Diverticular disease of colon	1.0 (0.4 - 1.6)		
**40**	Other endocrine disorders	1.0 (0.4 - 1.6)		
**41**	Substance use	1.0 (0.4 - 1.6)		

COPD, chronic obstructive pulmonary disease.

^1^excluding AMI.

^2^excluding diabetic retinopathy.

### Comorbidities extracted from multiple logistic regression analysis

Anxiety and neurosis in men (OR, 2.19; 95% CI, 1.84-2.60) and chronic skin ulcers in women (OR, 2.89; 95% CI, 2.38-3.53) were the diseases with the highest dementia-association probability. Of the 11 comorbidities found to be significantly associated with dementia, seven occurred in both men and women (anxiety and neurosis, Parkinson’s disease, chronic skin ulcers, anemia, cerebrovascular disease, cardiac arrhythmia and thyroid disease; Table 3).

**Table 3:** Odds ratios (OR) of dementia-associated chronic comorbidities in ≥65-year-old men and women

**Men**			**Women**		
**Disease**	**OR**	**CI 95%**	**Disease**	**OR**	**CI 95%**
Anxiety, neuroses	2.19	(1.84 - 2.60)	Chronic skin ulcers	2.89	(2.38 - 3.53)
Parkinson's disease	2.13	(1.49 - 3.06)	Anxiety, neuroses	1.79	(1.63 - 1.96)
Chronic skin ulcers	2.05	(1.41 - 2.96)	Anemia	1.57	(1.37 - 1.79)
Anemia	1.95	(1.58 - 2.41)	Cerebrovascular disease	1.57	(1.29 - 1.90)
Retinal disorders	1.72	(1.03 - 2.87)	Behavior problems	1.53	(1.22 - 1.92)
Cerebrovascular disease	1.63	(1.28 - 2.07)	Congestive heart failure	1.42	(1.15 - 1.75)
Cardiac arrhythmia	1.53	(1.25 - 1.88)	Parkinson's disease	1.41	(1.02 - 1.94)
Thyroid disease	1.43	(1.07 - 1.91)	Cardiac arrhythmia	1.24	(1.06 - 1.45)
Prostatic hypertrophy	1.29	(1.11 - 1.50)	Thyroid disease	1.17	(1.02 - 1.35)

### Exploratory factor analysis

**Figure 1:**
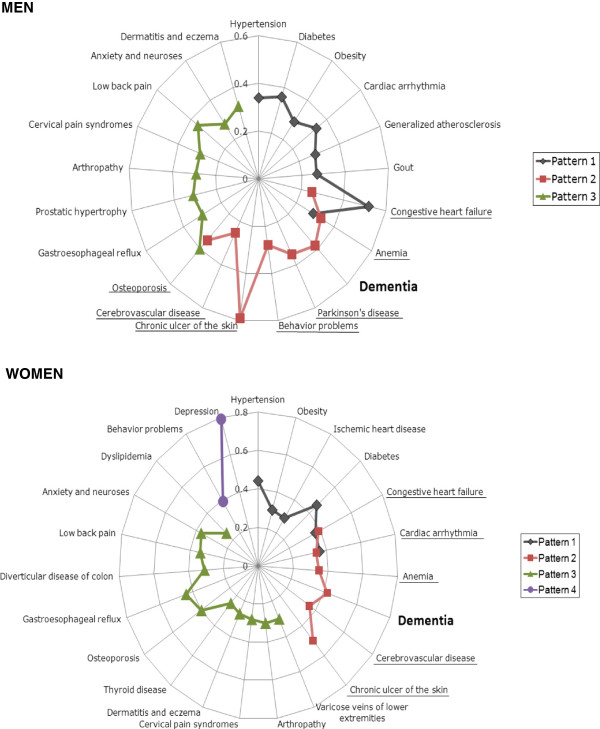
**Disease patterns and factor scores in ≥65-year-old men and women.***NOTES*: Each spoke of the radar chart represents a disease, and the lines connecting these spokes represent disease factor scores within each pattern. Only diseases with scores higher than 0.25 are shown. Underlined diseases belong to the pattern that includes dementia, and represent potential comorbidities of this index disease. KMO: men 0.57; women: 0.68. % Accumulated variance: men 22.81; women: 24.68.

The factor analysis for the whole population over 64 allowed the identification of three disease patterns in men, formed by an average of eight diseases. Dementia was present in only one pattern (Pattern 2) along with congestive heart failure, anemia, Parkinson’s disease, behavioral problems, chronic skin ulcers, cerebrovascular disease and osteoporosis (Figure 1).

Four disease patterns were identified in women, formed by an average of six diseases. As with men, dementia was present only in a single pattern (Pattern 2) associated with congestive heart failure, cardiac arrhythmia, anemia, cerebrovascular disease and chronic skin ulcers (Figure 1).

Therefore, only four diseases were identified (congestive heart failure, anemia, chronic skin ulcers and cerebrovascular disease) as forming part of the pattern that included dementia in both men and women.

Regarding the comparison with results obtained through logistic regression, both methods agreed on the identification of four comorbidities in men (Parkinson’s disease, chronic skin ulcers, cerebrovascular disease and anemia) and five comorbidities in women (chronic skin ulcers, cerebrovascular disease, anemia, congestive heart failure and cardiac arrhythmia).

## Discussion

This study showed that individuals over 64 with dementia have a significantly higher number of comorbidities than those not diagnosed with this index disease, thus confirming previous findings [8,11]. However, this could be due to the older age of patients with dementia. Among the multiple chronic comorbidities of dementia, only 12 seem to be significantly associated in the study population (Parkinson’s disease, congestive heart failure, cerebrovascular disease, anemia, cardiac arrhythmia, chronic skin ulcers, osteoporosis, thyroid disease, retinal disorders, prostatic hypertrophy, insomnia and anxiety and neurosis).

The main strength of the study lies in the data source used. The background is a primary care population served by a number of healthcare centers where diagnoses are systematically computer-stored for each patient. Moreover, the public nature of the healthcare system and high access of citizens, as well as the one-year observation period guarantee that selection bias is reduced. Still, our study is not free from limitations. The most important one concerns the cross-sectional study design; although this design allows for the generation of hypotheses regarding the clinical complexity of dementia, it does not allow for the determination of causality between the identified associations. Thus, no distinction can be made among risk factors, complications and/or simple comorbidities of dementia. There is evidence that dementia is under-diagnosed by general practitioners [24]. Regarding the way dementia was defined, no distinction was made between the different etiological types of dementia, and there can be differences between the comorbidities associated with each one of them [8]. It was neither possible to take into account the severity of dementia, which is relevant to the existence of possible comorbidities associated with functional dependence in these patients. Regarding the analyzed comorbidities, some non-chronic and/or unspecific conditions (falls, immobilization, etc.) that can also affect the quality of life of patients could not be included.

The two most frequent comorbidities both for men and women were hypertension and diabetes, a fact in disagreement with the results obtained through multivariate methods. Indeed, neither logistic regression nor factor analysis showed significant association between the aforementioned diseases and dementia, as has been shown previously [7,8]. Although hypertension and diabetes are risk factors for dementia in middle-aged populations, this association disappears in the elderly population [25]. Furthermore, in the specific case of hypertension, various longitudinal population-based studies have suggested that the relationship between hypertension and cognitive decline is not linear [26,27]. In summary, it is likely that the high prevalence of hypertension can be explained by its frequency rather than by the existence of common underlying pathogenic mechanisms [17]. However, in the case of diabetes, there is recent knowledge on the shared nature of type 2 diabetes and neurodegenerative and arteriosclerotic disorders associated with misfolded protein deposits [28]. These findings put forward the need to reinforce the convergence between basic, clinical and epidemiologic research.

On the other hand, logistic regression and factor analysis identified different chronic diseases significantly associated with dementia (Parkinson’s disease, congestive heart failure, cerebrovascular disease, anemia, cardiac arrhythmia, chronic skin ulcers, osteoporosis, thyroid disease, retinal disorders, prostatic hypertrophy, insomnia and anxiety and neurosis). For most of these comorbidities, a plausible pathophysiological explanation can be found [11]. Some could be considered as risk factors (cerebrovascular disease), others as complications (skin ulcers), and others just as comorbidities (osteoporosis). Alzheimer’s disease, preexisting vascular disease and Parkinson’s disease are among the most frequent etiologies of dementia [29,30]. Functional limitations and the inherent complications and risks associated with dementia and advanced Parkinson’s disease (e.g., falls) along with age-related osteoporosis can lead to fractures, patient immobilization and chronic skin ulcers, most likely associated with malnourishment and bedding in these patients [31]. Anemia and cardiac arrhythmia (mostly in the form of atrial fibrillation) are, in turn, causes of cerebrovascular disease [32]. Surprisingly, Zuliani et al. [31] and Sanderson et al. [8] identified a significant decreased risk of developing anemia and congestive heart failure among patients with dementia, but these findings refer to hospitalized patients who were most likely receiving effective treatments against both comorbidities, therefore weakening the possible association between dementia and such comorbidities.

Other studies that used factor analysis have also identified disease patterns that included dementia. Newcorner et al. [33] found a pattern they called “frailty in the elderly” among adult individuals in the United States. In addition to dementia, this pattern was characterized by the presence of skin ulcers, ictus, mental health problems and heart disease, among others. In a study conducted in Germany by Schäfer et al. [34], a neuropsychiatric pattern was described in which dementia appeared simultaneously with heart failure, Parkinson’s disease, depression, urinary incontinence, anemia and ictus, among other diseases. Albeit the existence of certain methodological differences, both studies show similar dementia comorbidity patterns as the ones obtained here.

Results of this study illustrate the pros and cons of certain statistical methods for the epidemiologic study of comorbidities of an index diagnosis. Frequency-based techniques are determined by the prevalence rates of each disease in the combinations and therefore have limited value. For example, given its high prevalence in the population, hypertension is one of the most frequent comorbidities of dementia. Thus, it is more informative to view comorbidities of an index disease from the perspective of the non-random association of health problems, as defined by the term associative comorbidity [16]. Odds ratios, which are widely used to identify associations between pairs of diseases [35], do not allow for the study of the simultaneous presence of diverse comorbidities, nor do they enable the exploration of possible associations between diseases that have not been established a priori. Moreover, such measures of association do not adequately adjust for chance comorbidity when non-random comorbidity exists [35]. Factor analysis allows the visualization of disease clustering into patterns, offering results of aetiological interest [19]. In some studies, cluster instead of factor analysis was applied [36,37], but this technique does not allow for health problems to simultaneously belong to more than one cluster. Other alternatives include the use of comorbidity scores, which incorporate available diagnostic information into an aggregate index to trace older patients who are at high risk for hospitalization or mortality [38,39]. However, these measures preclude estimations of effects of individual or groups of disease [13].

A deep and reliable knowledge about how different diseases are grouped and associated may orient future longitudinal studies aimed at unraveling causal associations [40]. Moreover, the identification of eventual comorbidity patterns may facilitate diagnosis and effective treatment of comorbidities among individuals suffering from a given index disease. Last, knowledge regarding the potential of such patterns as predictors of intense healthcare use, adverse health outcomes (e.g., disability, adverse drug events) and/or increased severity of a given disease could be incorporated into risk stratification tools [41].

## Conclusion

The analysis of the comorbidities associated with an index disease (e.g., dementia) must not be exclusively based on prevalence rates, but rather on methodologies that allow the discovery of non-random associations between diseases. A deep and reliable knowledge about how different diseases are grouped and associated around an index disease such as dementia may orient future longitudinal studies aimed at unraveling causal associations.

## Competing interests

There are no conflicts of interest for any of the authors.

## Authors’ contributions

BPP, ACL and APT conceived the study. BPP and JHS undertook the statistical analysis and all authors were involved in the interpretation of the data. BPP, ACL, JHS and APT wrote the first draft of the paper. All authors revised it critically for important intellectual content and approved the final version.

## Pre-publication history

The pre-publication history for this paper can be accessed here:

http://www.biomedcentral.com/1471-244X/14/84/prepub
